# Neuronal contact guidance and YAP signaling on ultra-small nanogratings

**DOI:** 10.1038/s41598-020-60745-5

**Published:** 2020-02-28

**Authors:** Ilaria Tonazzini, Cecilia Masciullo, Eleonora Savi, Agnese Sonato, Filippo Romanato, Marco Cecchini

**Affiliations:** 1 NEST, Istituto Nanoscienze-CNR and Scuola Normale Superiore, Piazza San Silvestro 12, Pisa, 56127 Italy; 2 CNR-IOM, Area Science Park, S.S. 14, km 163.5, Basovizza, TS Italy

**Keywords:** Biotechnology, Biomaterials, Biomaterials - cells

## Abstract

Contact interaction of neuronal cells with extracellular nanometric features can be exploited to investigate and modulate cellular responses. By exploiting nanogratings (NGs) with linewidth from 500 nm down to 100 nm, we here study neurite contact guidance along ultra-small directional topographies. The impact of NG lateral dimension on the neuronal morphotype, neurite alignment, focal adhesion (FA) development and YAP activation is investigated in nerve growth factor (NGF)-differentiating PC12 cells and in primary hippocampal neurons, by confocal and live-cell total internal reflection fluorescence (TIRF) microscopy, and at molecular level. We demonstrate that loss of neurite guidance occurs in NGs with periodicity below 400 nm and correlates with a loss of FA lateral constriction and spatial organization. We found that YAP intracellular localization is modulated by the presence of NGs, but it is not sensitive to their periodicity. Nocodazole, a drug that can increase cell contractility, is finally tested for rescuing neurite alignment showing mild ameliorative effects. Our results provide new indications for a rational design of biocompatible scaffolds for enhancing nerve-regeneration processes.

## Introduction

With its chemical and physical complexity, the extracellular environment can drive cell morphogenesis, migration and differentiation^1–4^, In particular, the contact interaction of cells with extracellular nanometric features has a primary role in regulating many physiological^5–12^ and pathological processes^13^ *in vivo*, and can be exploited to modulate cell responses *in vitro*^14–17^. Cells gather morphological information about the Extracellular matrix (**ECM**) through integrin-mediated adhesion clusters, called the focal adhesions (**FAs**), which act as topographical sensors. FAs can integrate multiple nanotopographical details into specific biomolecular instructions via the cytoskeletal signaling, which lastly regulates cell contractility, morphology and migration^9,18,19^,

Artificial scaffolds with controlled micro/nano-topographies are definitively the apparatus of choice to better understand the response mechanisms of cells to external stimuli. They indeed allow to select and finely tune specific topographical aspects and selectively study their interaction with living system *in vitro*. The scientific literature reports many examples of nanostructures that can, for example, direct stem cell fate^20–22^, neuronal and glial adhesion^23–25^, differentiation^26^, polarization, and neurite orientation^14,18^, Our previous studies have demonstrated that plastic nanogratings (**NGs**) (i.e. alternating lines of sub-micron grooves and ridges, in the range between 500 and 2000 nm in linewidth) can promote neurite alignment and bipolarity of PC12 neuronal cells upon administration of nerve grow factor (**NGF**), simply by the contact guidance mechanism^27,28^, In these studies, adhesion on the ridges imposes a geometrical and directional constraint to FAs that results in neuronal polarization via the ROCK-mediated pathway^14,29–31^, The best results for PC12 neurite alignment and bipolarization were obtained with NGs having 1 μm periodicity (i.e. 500 nm linewidth)^14^.

Nanotopographies can thereby tailor the cell phenotype and several morpho-functional aspects, with obvious advantages for tissue engineering and regeneration applications. However, in literature there are only a few studies about cellular behavior in response to NGs with lateral period smaller than 1 μm. In the case of T-cells on 500 nm-period NGs, enhanced proliferation was reported, but contact guidance was ineffective^32^. Similarly, fibroblasts lost topographical guidance both along 600 nm-period PDMS^33^ and 100 nm-period polystyrene (PS) nano-grooved substrates^34,35^ cardiomyocyte did the same on 600 nm-period PCL NGs^36^, while rat glioma C6 cells exhibited directional migration and oriented division along 266 nm-period PS NGs^37^. PDMS NGs with lateral periodicity of 700 nm enhanced differentiation of human mesenchymal cell into a neuronal lineage^38^. Furthermore, the cell alignment switched from parallel to perpendicular when human corneal epithelial were cultured on silicon NGs with periodicity decreasing from 4 um to 400 nm^39^, and similarly Schwann cells showed a perpendicular alignment on PET 300 nm–period nano-ripples^23^. Finally, silicon 400 nm-periodic NGs reduced neuritogenesis but were effective in aligning rat pheochromocytoma (PC12) cells^40^.

Recent studies have emphasized the involvement in mechano-sensing of Yes-associated protein (**YAP**) transcription factor^41,42^ coupled with its transcriptional coactivator with PDZ-binding motif (**TAZ**), with a crucial role in correlating external mechanical stimuli with changes in gene expression^43^. More specifically, changes in ECM stiffness can determine modifications in cytoskeleton organization and tension, shuttling the YAP/TAZ complex to the nucleus^44^ and influencing the mechanotransduction process at FA level^45^, in a feedback loop between cytoskeleton and nucleus. Nowadays, the influence of substrate stiffness on YAP localization is known^44^, but the effect of nanotopography on the activation and intracellular localization of YAP has not been deeply explored yet. At the best of our knowledge, only a few studies are reported in literature. Human induced pluripotent stem cells (hiPSCs) showed higher YAP cytoplasmic localization (i.e. lower activation) on PDMS NGs (500 nm linewidth, 1000 nm periodicity, 560 nm depth) compared to flat controls^46^. On 200 nm-diameter Platinum Bulk Metallic Glass (Pt-BMG) nanorods (with an height ≅ 2 μm) human MSC spreading was reduced, leading to the cytoplasmic translocation and inactivation of YAP/TAZ^47^. In another study, randomly shaped nanotopographies with typical dimension of the order to few hundreds of nm regulated YAP phosphorylation and nuclear shuttling in human pluripotent stem cells (hPSCs)^48^. On the contrary, arrays of NOA81 polymeric NGs with lateral periodicity ranging from 400 nm to 4 μm (50% duty cycle, 300 nm height) did not alter the spatial organization and expression of YAP in human corneal epithelial cells^49^. The localization of YAP was also pattern-independent in human embryonic stem cells cultured on both NGs (250 nm linewidth, 500 nm periodicity, 250 nm depth) and unpatterned PDMS substrates^50^, and also in MSCs cultured on polyamide substrates (flat or NG topography with 650 nm linewidth, 1300 nm periodicity, 200 nm depth)^51^. A very recent study showed an enhanced substrate epithelialization on grating substrates (1 µm linewidth and depth) demonstrating a positive correlation between YAP/TAZ nuclear localization and the wound healing efficacy^52^. Finally, the effect of nanostructured ridge arrays (800 nm width and depth) on epithelial-to-mesenchymal transition was showed to be mediated by YAP as well^53^.

Here, we report on neuronal contact guidance on ultra-small NGs. Substrates with lateral period from 1000 nm (considered as a reference substrate owing to its known ability to exert excellent contact guidance) down to 200 nm were fabricated in cyclic olefin copolymer (COC) by a two-step nanoimprinting process starting from molds produced by electron beam or laser interferometric lithography. The impact of ultra-small NGs on cell morphotype, neurite alignment, focal adhesion (FA) development and YAP activation is investigated in NGF-differentiating PC12 cells by confocal and live-cell total internal reflection fluorescence (TIRF) microscopy, and at molecular level. We also validate the impact of ultra-small NGs on primary neuron guidance. Finally, we explore the use of blebbistatin and nocodazole, two pharmacological treatments targeting cell contractility, to tune and recover the guidance where less effective.

## Results

### Ultra-small thermoplastic nanogratings

We developed ultra-small nanograting (NG) topographies (i.e. anisotropic patterns of alternating lines of ridges and grooves) with the aim of studying the interaction between cells and anisotropic features with controlled nanometric periodicity, towards the typical dimensions of ECM cues. To this end, we fabricated a complete set of NGs with 50% duty cycle (i.e. ridge width/period = 0.5) and period 1 µm (named T1, groove depth = 350 nm), 600 nm (named T600, groove depth = 300 nm), 400 nm (named T400, groove depth = 200 nm) and 200 nm (named T200, groove depth = 100 nm). Given our previous results^40^, the T1 geometry is here considered as the gold standard guidance topography for our neuronal cell model.

**Figure 1:**
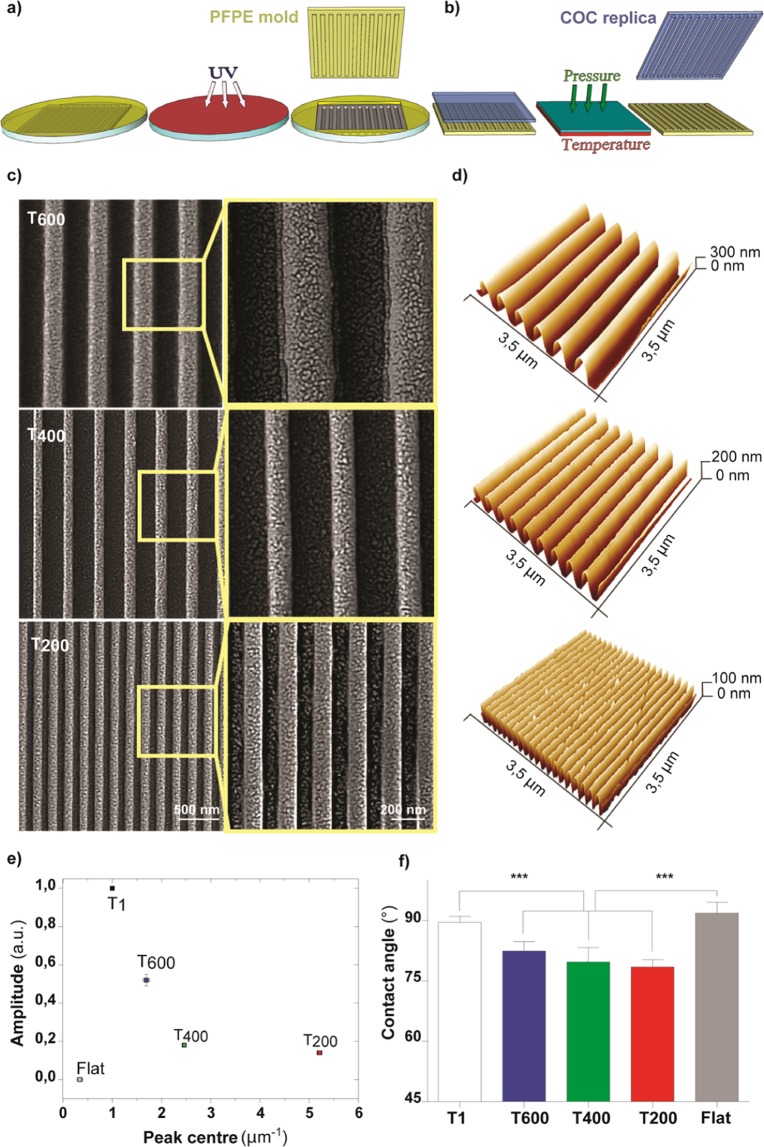
Ultra-small nanograting fabrication scheme and surface characterization. (**a**) PFPE intermediate mold (*in yellow*) fabrication via UV-crosslink process and (**b**) its use for the subsequent thermal NIL process to produce Cyclic Olefin Copolymer (COC) replicas (*in light blue*). (**c**) Representative Scanning Electron Microscopy images (with high magnification insets) of the COC NGs T600, T400 and T200. (**d**) AFM measurements of NG final COC replica, for 3.5 × 3.5 μm^2^ areas. (**e**) NG directionality signal amplitude by FFT analysis: the reported values were normalized to T1 value. ***P < 0.001 T1 *vs*. T600, T400, T200 and T600 *vs*. T400, T200; *P < 0.05 T400 *vs*. T200; One-way ANOVA Tukey’s test. (**f**) Contact angle measurements of COC NGs. ***P < 0.001 T1 *vs*. T600, T400, T200 and Flat *vs*. T600, T400, T200 (One-way ANOVA, Tukey’s test). All data are mean ± SD.

NG patterns were replicated from initial molds into biocompatible and transparent Cyclic Olefin Copolymer (COC) thermoplastic polymer foils by exploiting perfluoropolyether (PFPE) intermediate molds, as described in Materials and Methods and in^54^. Briefly, PFPE intermediate molds were obtained by pouring a PFPE dispersion on top of each initial mold, and then crosslinking with UV light (Fig. 1a). The final NG replicas were produced via the PFPE molds into COC films by Nanoimprint Lithography (Fig. 1b).

As shown in Fig. 1c, both ridges and grooves retained their width after the two replica processes. In particular, ridges were laterally squared and straight, thus maintaining the grating initial profile and dimensions. NGs did not present discontinuities as holes or cracks along the ridges, thanks to the correct calibration of NIL process in terms of working temperature and applied pressure. Moreover, the use of a soft mold instead avoided nano-feature disruption during NIL, ensuring the nanopattern continuity on the whole patterned area.

The nanopatterns were further characterized by AFM measurements (Fig. 1d), allowing us to calculate their aspect ratio (defined as the ratio of ridge height over width). We measured the following periodicities: 610 ± 20 nm for T600 (ridge width = 300 ± 30 nm, ridge height = 300 ± 20 nm, aspect ratio = 1.0 ± 0.1); 400 ± 10 nm for T400 (ridge width: 210 ± 20 nm, ridge height: 200 ± 20 nm, aspect ratio = 1.0 ± 0.2); 200 ± 10 nm for T200 (ridge width: 110 ± 20 nm, ridge height: 95 ± 15 nm, aspect ratio = 0.9 ± 0.2). As reference, T1 showed 1000 ± 30 nm periodicity (ridge width: 505 ± 25 nm, ridge height 350 ± 30 nm, calculated aspect ratio 0.7 ± 0.1; (all dimensions are mean ± SD, calculated using at least ten profiles extracted from 3.5 µm × 3.5 µm AFM measured area for each NGs).

By an FFT analysis on the AFM profiles we estimated the directionality of the different NGs (*see Materials and Methods* for details). Directionality amplitudes (normalized to the value measured for T1) were 0.52 ± 0.03 for T600 (peak centered at 1.67 ± 0.05 µm^−1^), 0.19 ± 0.01 for T400 (peak centered at 2.43 ± 0.01 µm^−1^), 0.14 ± 0.01 for T200 (peak centered at 5.14 ± 0.01 µm^−1^) and 0.01 ± 0.01 for the flat substrate (peak centered at 0.34 ± 0.06 µm^−1^) (Fig. 1e). All NGs showed a higher directionality if compared to flat surfaces (P < 0.001 T1-T600-T400-T200 *vs*. Flat, Tukey’s test), as expected. Among the different NGs, we found an increasing trend with the period (P < 0.001 T1 *vs*. T600, T1 *vs*. T400, T1 *vs*. T200; P < 0.001 T600 *vs*. T400, T600 *vs*. T200; P < 0.05 T400 *vs*. T200, Tukey’s test).

Given that a good surface hydrophilicity generally promotes cell adhesion and spreading^21,24^, in order to further characterize the substrate surface properties, we evaluated water wettability by performing water contact angle measurements (Fig. 1f). As shown in Fig. 1f, T600, T400 and T200, showing contact angles of 82 ± 2°, 80 ± 3° and 78 ± 1° respectively, were more hydrophilic than the T1 and Flat (contact angles of 89 ± 2° and 95 ± 3°; P < 0.001, Tukey’s test).

In summary, we fabricated biocompatible thermoplastic NGs with lateral periodicity ranging from 1 µm down to 200 nm, with aspect ratio between 0.7 (T1) and 1 (T600, T400 and T200). We replicated these nanostructures by a thermal embossing process, using PFPE intermediate molds to preserve the original molds and enhance the fabrication process yield. For all ultra-small NGs, the topographies were preserved during the entire two-step replica process.

#### Neurite contact guidance

**Figure 2:**
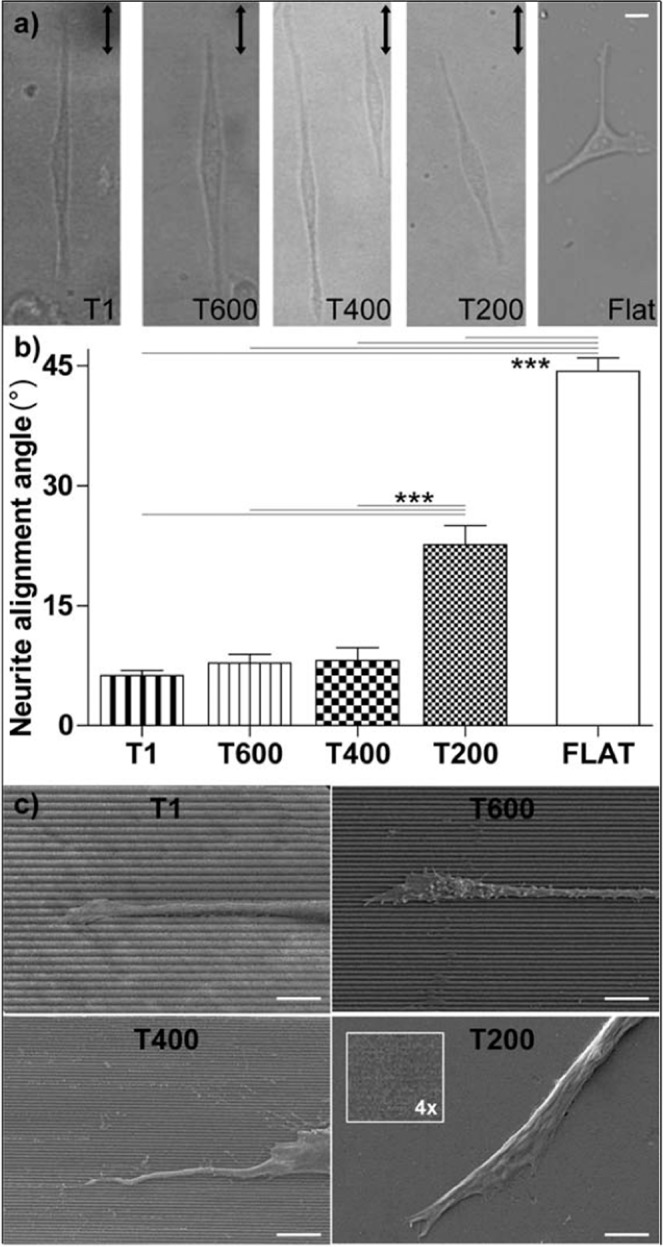
Neurite alignment along NGs. (**a**) Bright-field images of PC12 neuronal cells on T1, T600, T400, T200 and Flat, at 24 h; the arrows indicate the NG direction; scale bar = 10 μm. (**b**) Neurite alignment on NGs. ***P < 0.001 (One-way ANOVA, Tukey’s test); at least 300 cells –at least 450 neurites- were analyzed for each substrate and for each NG type we performed n ≥ 6 independent experiments. (**c)** Representative scanning electron microscopy (SEM) images of PC12 neurites on T1, T600, T400 and T200; scale bars = 5 μm.

In order to assess the neuronal guidance on NGs with very small periodicity, PC12 cells were seeded on the complete set of NGs (periods from 1 µm down to 200 nm), and neuronal differentiation was induced by NGF stimulation (100 ng/ml) (Fig. 2a). The emission of neuronal protrusions (neurites) was influenced by the local substrate nanotopography, and we quantified the cell alignment by measuring neurite alignment (the average angle between neurites and NG direction) such as neurite morphological parameter – at t = 24 h by bright-field optical microscopy (Fig. 2a). As expected, neurites interacting with T1 extended along the pattern direction (neurite alignment = 6 ± 1°). Neurite alignment is maintained on T600 (8 ± 1°) and T400 (8 ± 2°) (P>0.05 *vs*. T1, P < 0.001 *vs*. Flat; Tukey’s test); instead we observed a significant alignment loss by decreasing the period down to 200 nm (23 ± 2°) (P < 0.001 T200 *vs*. T1/T600/T400; Tukey’s test) (Fig. 2b). However, T200 directionality could still be retrieved by PC12, inducing a partial neurite alignment if compared to the random neuritic distribution that was measured on Flat substrates (44 ± 2°; P < 0.001 T200 *vs*. Flat; Tukey’s test). We also quantified neurite length (Fig. 2a). Neurite length was not affected by the reduction of NG periodicity (Fig. S1). Scanning electron microscopy images showed in details the interaction between PC12 neurites and NG topographies (Fig. 2c).

These results indicate that cell and neurite alignment of NGF- differentiated PC12 cells is sensitive to ultra-small NGs and their directionality signal, despite neurite guidance is impaired on T200 substrates.

#### Focal adhesion maturation is modulated by ultra-small nanogratings

We previously showed that in PC12 cells the induction and growth of neurites along 1-µm-period gratings were critically controlled by the establishment and maturation of FAs^14,29,30^ here, in fact, FAs were squeezed and grew directed along ridges. Ultra-small NGs were also expected to importantly affect FAs because their typical length-scale still is of the order of that of ECM protein clusters^9^.

**Figure 3:**
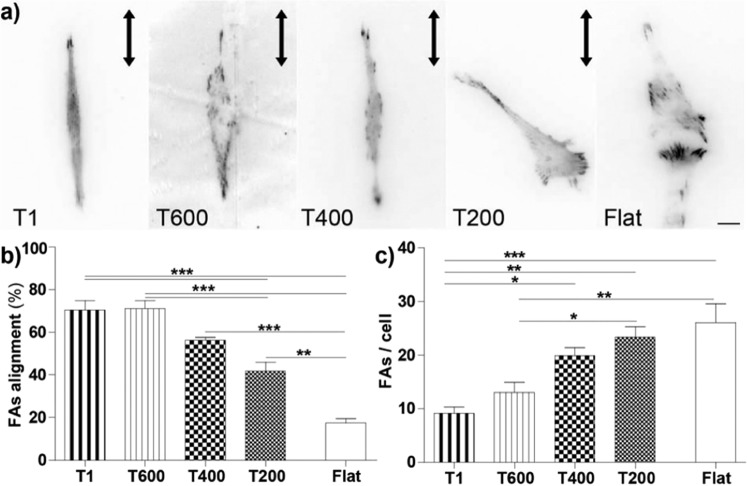
Impact of reduced NG periodicity on FA assembly and spatial distribution. (**a**) EGFP-Paxillin rich adhesions on NGs imaged by TIRF microscopy; scale bar = 10 µm. (**b**) FA alignment on different NGs, reported as the % of FAs with alignment ≤ 15°. **/***P < 0.01/0.001 (One-way ANOVA, Tukey’s test). (**c**) Number of FAs per cell on different substrates. *P < 0.05, **P < 0.01 and ***P < 0.001 (One-Way ANOVA, Tukey’s test); at least 30 cells or 400 FAs were analyzed for each sample and for each NG type we performed n ≥ 4 independent experiments.

In order to validate this hypothesis, PC12 cells were transiently transfected with EGFP-Paxillin, a signal-transduction protein present in early and mature FAs^9^, and differentiated by NGF on the ultra-small NGs. Paxillin was then selectively visualized at the cell basal-membrane by live cell TIRF microscopy (Fig. 3a) and FAs were analyzed by measuring their alignment (°) with respect to the NG direction **(**Fig. 3b) and quantifying their number per cell (Fig. 3c). The progressive reduction of NG periodicity induced a decrease in the overall *percentage of FAs alignment*, reported here as the percentage of FAs with an alignment angle ≤ 15° vs. NG direction **(**Fig. 3b), as we already found for neurite guidance (Fig. 2b). FA alignment started to be compromised on T400 (56 ± 1%) and even further on T200 (42 ± 4%). The percentage of FA alignment was indeed significantly lower if compared to that measured for T1 (70 ± 4%) and T600 (71 ± 4%) (P < 0.001 T200 *vs*. T1 and T600, Tukey’s test). However, T400 and even T200 could still partially polarize the FA development along NGs in comparison to isotropic Flat substrates (17 ± 2%) (P < 0.001 T400 *vs*. Flat, P < 0.01 T200 *vs*. Flat; Tukey’s test; Fig. 3b).

The decrease of NG periodicity also induced a progressive increase in the number of FAs/cell (Fig. 3c). Interestingly, the number of FAs developed by each cell increased from 9 ± 1 on T1 and 13 ± 2 on T600 to 20 ± 1 on T400 and 23 ± 2 on T200, with values similar to those obtained for the Flat surface (26 ± 3) (P < 0.05/P < 0.001 T1 *vs*. T400, T200 and Flat; P < 0.05/0.01 T600 *vs*. T200 and Flat; Tukey’s test, Fig. 3c). Overall, FA spatial organization and number per cell were progressively affected by decreasing the period of ultra-small NGs, well correlating with neurite guidance. T400 behaved such as a “border land” substrate between FA alignment and misalignment regimes.

**Figure 4:**
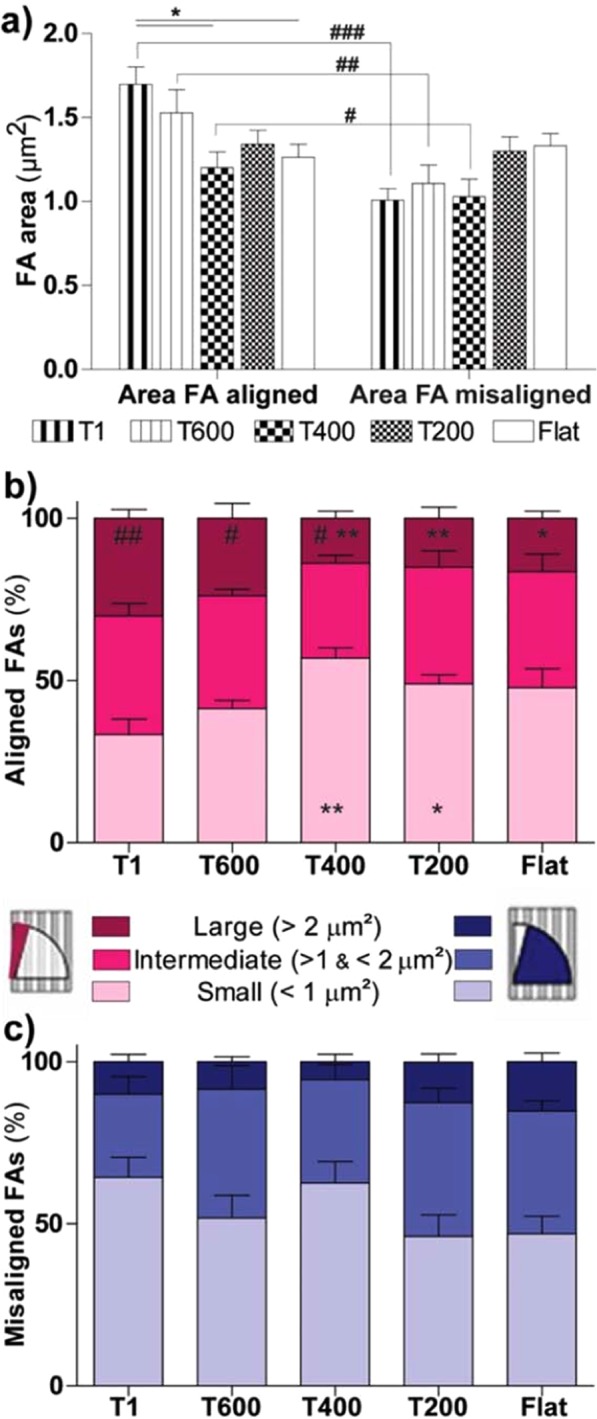
Impact of reduced substrate dimensionality on FA maturation. (**a**) Average FA area (µm^2^) is reported for *aligned* FAs (i.e. with alignment ≤ 15°; *left panel*) and for *misaligned* FAs (i.e. with alignment between 15° and 90°; *right panel*), on the different substrates. Aligned FA area *vs*. misaligned FA area: ^###^P < 0.001, ^##^P < 0.0, ^#^P = 0.05 (Student’s t-test). Aligned FA area: *P < 0.05 T1 *vs*. T400 and Flat (One-Way ANOVA, Tukey’s test). (**b**) Distribution of FA size NGs. Size distribution of aligned (0–15°) FAs, as function of the substrate. The % of small (area ≤ 1 µm^2^), intermediate (1 < area ≤ 2 µm^2^) and large (area > 2 µm^2^) FAs is reported in *light, normal* and *dark* pink color, respectively. Small FAs: */**P < 0.05/0.01 *vs*. T1; Large FAs: */**P < 0.05/0.01 *vs*. T1; (One-Way ANOVA, Tukey’s test). **c**) Size distribution of misaligned (15–90°) FAs as function of the substrate. The % of small (area ≤ 1 µm^2^), intermediate (1 < area ≤ 2 µm^2^) and large (area > 2 µm^2^) FAs is reported in *light, normal* and *dark* blue color, respectively. Large-Aligned FAs (%) *(darker pink columns*-in (**b**) *vs*. Large-Misaligned FAs (%) *(darker blue columns*- in (**c**): ^##^P < 0.01 for T1, ^#^P < 0.05 for T600 and T400, Student’s t-test. At least 30 cells or 400 FAs were analyzed for each sample and for each NG type we performed n ≥ 4 independent experiments.

FA maturation was then analyzed on the different substrates, by measuring the FA size (Fig. 4). FAs were classified as *aligned* (in case of alignment angle ≤ 15°) or *misaligned* (in case of alignment angle > 15°). Even if the overall average FA size did not show major changes by reducing the NG periodicity (Fig. S2), the maturation (i.e. size) of the aligned FA was progressively affected by the reduction of NG periodicity (Fig. 4a), progressively becoming indistinguishable from the misaligned ones. The size of aligned FAs was larger on T1 (i.e. the substrate with the best neurite guidance behavior, 1.7 ± 0.1 μm^2^) with respect to those developing on substrates with less neurite-guidance abilities, in particular on T400 (1.2 ± 0.1 μm^2^) and Flat (1.3 ± 0.1 μm^2^) (for Aligned FAs: P < 0.05 T1 *vs*. T400 and Flat, Tukey’s test; Fig. 4a-*left panel*). Moreover, aligned FAs were larger than the misaligned ones (Fig. 4a-*right panel*) for cells on T1 (1.7 ± 0.1 μm^2^ and 1.0 ± 0.1 μm^2^, respectively) (P < 0.001, Size FA Aligned *vs*. Misaligned on T1, Student’s t-test), T600 (1.5 ± 0.1 μm^2^ and 1.1 ± 0.1 μm^2^) (P < 0.01, Student’s t-test) and on T400 (1.2 ± 0.1 μm^2^ and 1.0 ± 0.1 μm^2^) (P = 0.05, Student’s t-test). Considering misaligned FAs, a general trend could not be identified although T200 and Flat showed the tendency to allow more effective maturation (Fig. 4a-*right panel*). In summary, these experiments demonstrated that the reduction of NG periodicity modulated FA maturation. T1 and T600 patterns could favor the maturation of aligned FAs while reducing misaligned-FA development. T400 represented the limit geometry where aligned FAs could develop larger in size than the misaligned ones, while on T200 they did not.

In order to better highlight this effect, all FAs were sorted in three categories (Fig. 4b,c): small (FA area ≤ 1µm^2^), intermediate (1 µm^2^ < area ≤ 2 µm^2^), and large (>2 µm^2^), corresponding to different maturation stages^29^. For aligned FAs, lowering NG periodicity leaded to an increase of the small-FA population (from ~33% on T1 to a maximum of 57% on T400; P < 0.01 T1 *vs*. T400 and P < 0.05 T1 *vs*. T200, Dunnett’s test), and reduction of large FAs (from ~30% to 14%; P < 0.01 T1 *vs*. T400 and T200, P < 0.05 T1 *vs*. Flat, Dunnett’s test; Fig. 4b). Interestingly, the number of large FAs developing aligned on T1, T600 and T400 was much greater than that of large misaligned FAs (#Aligned FAs (%)- Large *vs*. Misaligned FAs (%)-Large: P < 0.01 for T1, P < 0.05 for T600 and T400, Student’s t-test; Fig. 4b,c, *darker columns*). No evident trends were instead identified for misaligned FAs (Fig. 4c). These data demonstrate that differentiating PC12 cells actively respond to the reduction of NG periodicity through the variation of FA development and spatial maturation. Overall T400 represents a limit substrate to control the directional development of FAs and the subsequent neurite guidance.

**Figure 5:**
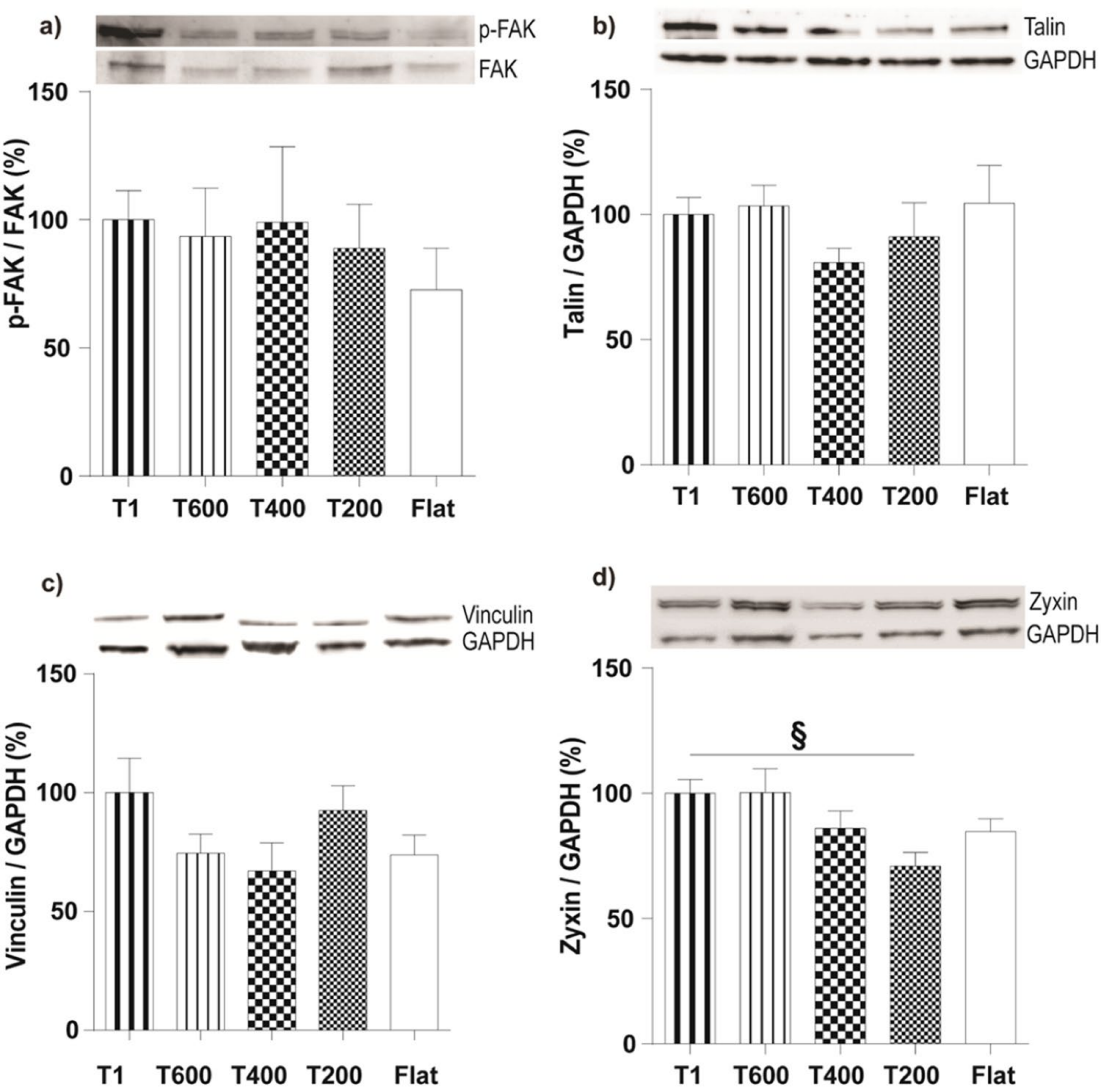
Activation of the FA pathway in PC12 cells growing on ultra-small NGs. Representative Western-blot panels (from the same gels; *full-length gels are included in* Fig. S3) and blot analysis of phospho-FAK/FAK (**a**), talin (**b**), vinculin (**c**), and zyxin (**d**) levels. Results (normalized to GAPDH levels) were reported in % with respect to T1 levels. d) *P < 0.05 T1 *vs*. T200 (One-Way ANOVA, Dunnett’s test); n ≥ 4.

Because FAs mediate neurite outgrowth and the integration of topographical information into cytoskeletal signaling^11^, we investigated the effects of ultra-small NGs on the FA pathway at molecular level, focusing on the activation of FA kinase (FAK), and on the levels of a set of FA scaffold proteins: Talin, Vinculin and Zyxin (Fig. 5). The activation of FAK was not affected by the NGs, as well as the levels of Talin and Vinculin (P > 0.05). The total levels of Zyxin, which is present only in mature FAs^9^, showed instead a decreasing trend by lowering the NG dimensionality, and it was significantly reduced on T200 (P < 0.05 T1 *vs*. T200, Dunnett’s test) (Fig. 5d).

#### Pharmacological tuning

**Figure 6:**
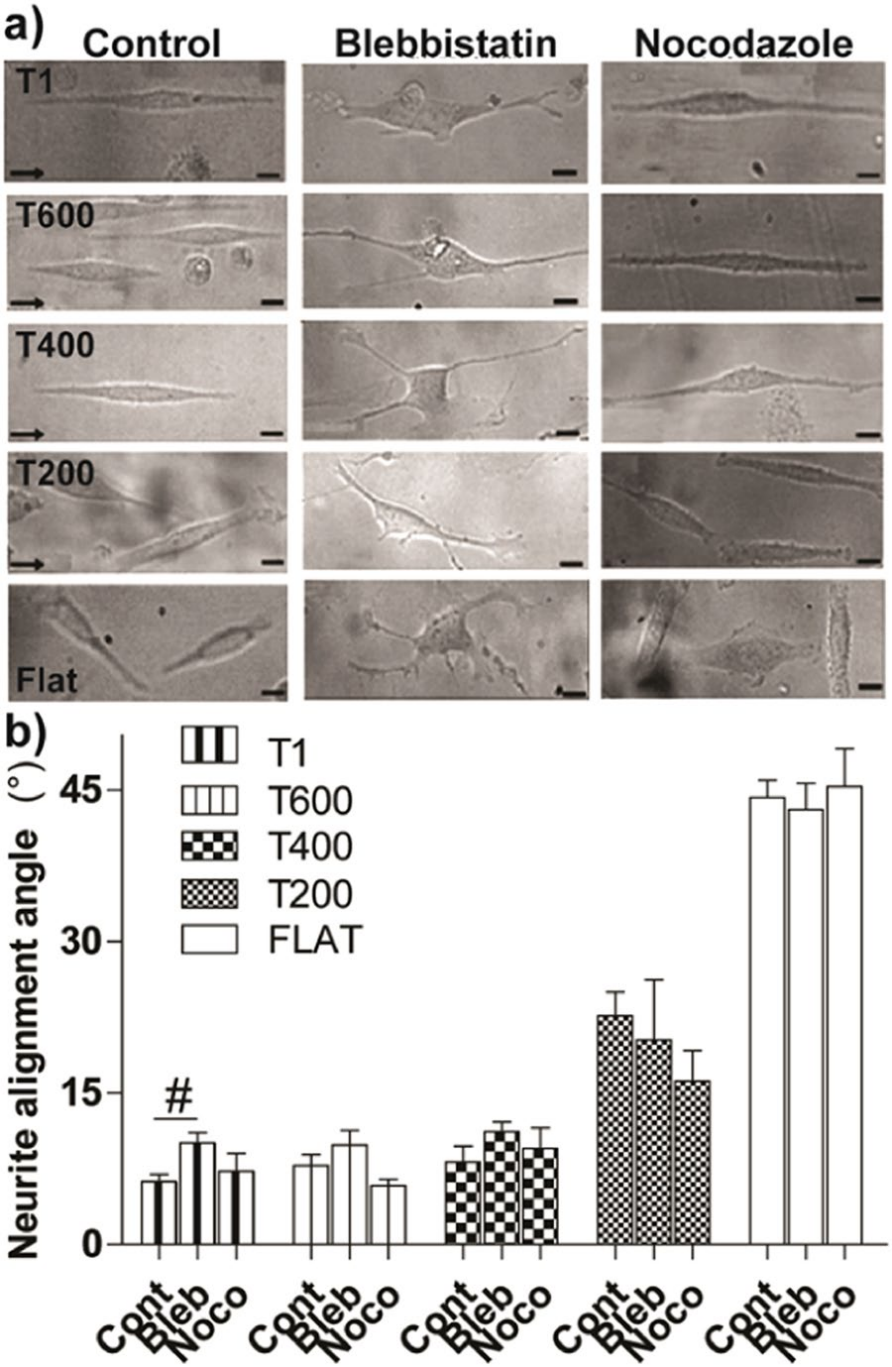
Neurite alignment along ultra-small NGs: role of cell contractility. (**a**) Bright-field images (*Top panel)* of PC12 neuronal cells on different NGs, in control conditions and in presence of blebbistatin 25 µM (Bleb) and nocodazole 10 nM (Noco); the arrows indicate the NG direction; scale bars = 10 μm. (**b**) Neurite alignment on NGs: ^#^P < 0.05 T1-Cont *vs*. T1-Bleb, Student’s t-test. Data are reported as mean ± SEM (At least 300 cells - 450 neurites- were analyzed for each substrate in control conditions (n ≥ 6), and 110 cells - 180 neurites- for each substrate with drugs’ treatments (n ≥ 3).

We previously demonstrated that PC12 contact guidance on NGs requires myosin-II/Rho-mediated contractility, and that the tolerance to topographical noise can be tuned by pharmacologically interfering with this signaling pathway^11^. We therefore exposed PC12 cells grown on ultra-small NGs to nocodazole (**Noco**), a microtubule depolymerizing agent that can activate the RhoA-mediated cell contractility and improve neurite contact guidance on noisy NGs, and to blebbistatin (**Bleb**), a myosin-II-contractility inhibiting drug, that was shown to impair neurite contact guidance^11,29^ (Fig. 6a).

In line with our previous results, Bleb treatment impaired neurite guidance along T1 (P < 0.05 T1-Ctrl *vs*. T1-Bleb, Student’s t-test), even if with no effects on smaller NGs. We then measured neurite alignment in presence of Noco: Noco had not detectable effect on the “well-guiding” NGs T1, T600 and T400. The average neurite alignment angle on T200 + Noco drops to 16 ± 3° (Fig. 6b), from the 23 ± 2° on T200 Ctrl, although this difference was not statistically significant (T200 Ctrl *vs*. T200 + Noco, P = 0.08). Neurite growth and length were not affected by both drugs’ treatments (Fig. S1).Beside the key role of the cell-contractility machinery in topographical guidance, Noco and Bleb had no major effects on neurite guidance on ultra-small NGs.

#### YAP signaling

**Figure 7:**
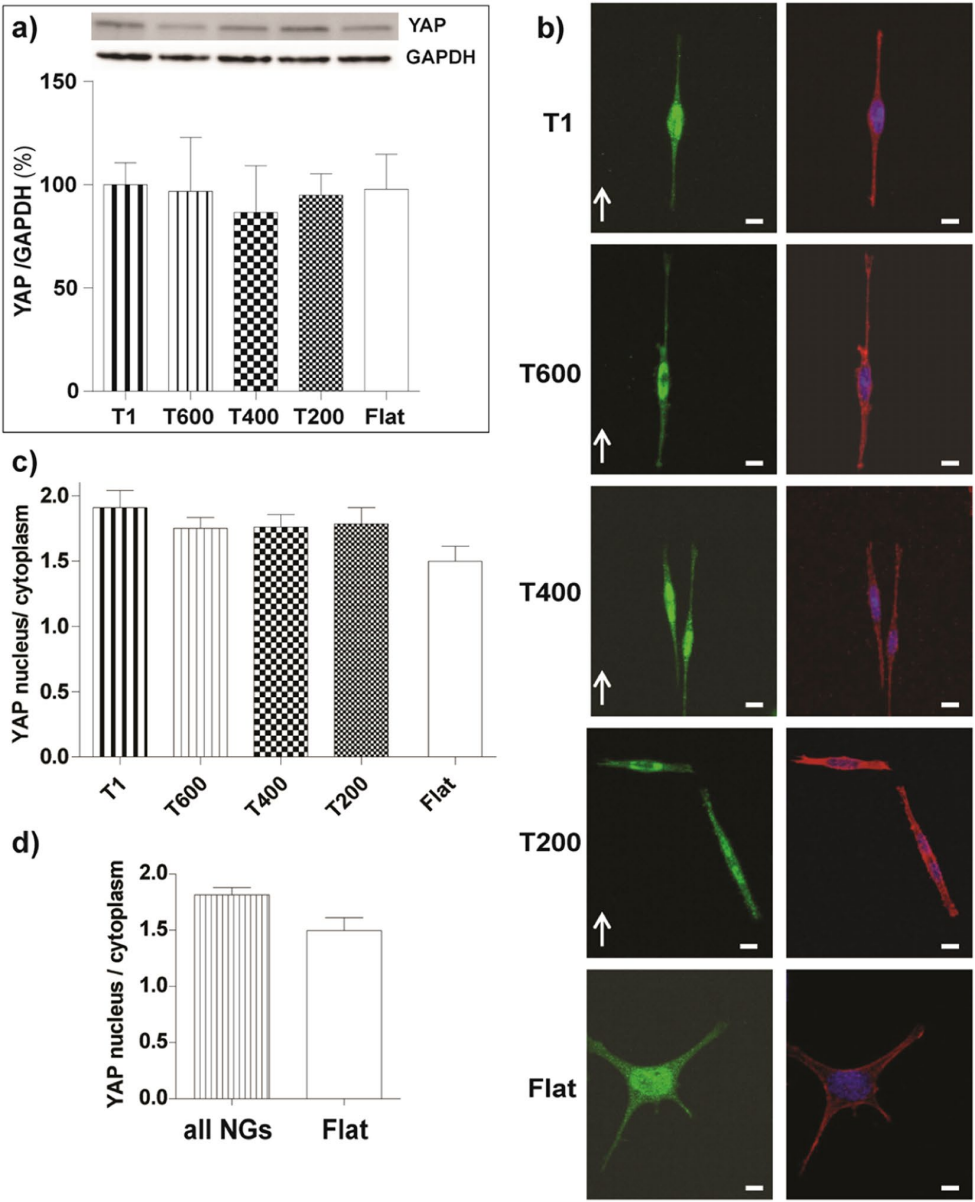
Activation of the YAP/TAZ pathway in PC12 cells differentiating on ultra-small NGs. (**a)** Representative Western-blot panels (from the same gel; *full-length gels are included in* Fig. S3) and blot analysis of YAP 1 expression levels. Results (normalized to GAPDH levels) were reported in % (with respect to T1 levels; n ≥ 3. (**b)** Confocal representative images of YAP (*green, first column*) and actin fibers (*red*) with nuclear (*blue*) staining in PC12 cells on different substrates; scale bars = 10 μm. (**c)** YAP intracellular localization: YAP activation is reported as YAP nuclear/cytoplasmic ratio on different substrates; at least 25 cells were analyzed for each sample (n ≥ 3). (**d)** YAP nuclear/cytoplasmic ratio on all NGs- pooled together and Flat surfaces: ^#^P < 0.01 NGs *vs*. Flat (Student’s t-test).

We finally investigated the involvement of YAP/TAZ signaling in neurite contact guidance on ultra-small NGs. As first, we measured the YAP level, which resulted unaffected by the presence of the nanotopography (Fig. 7a). Once activated, YAP concentrates into the nucleus. We thus performed immunostaining for YAP to investigate its intracellular localization (Fig. 7b), with the aim to test if the different guidance performance could be linked to different activation of the YAP/TAZ pathway. The nuclear/cytoplasm localization ratio was similar on the different NGs (Fig. 7c). Nevertheless, YAP was more activated and localized in the nucleus of cells cultured on all types of NGs pooled together (1.80 ± 0.04) with respect to those on Flat (1.5 ± 0.1, P < 0.01 NGs *vs*. Flat, Student’s t-test; Fig. 7d).

We finally tested the effects of drugs acting on cell cytoskeleton, Noco and Bleb, on YAP intracellular localization (Fig. S4a). We chose 3 different substrates: T600 as good-guidance substrate, T200 as impaired-guidance substrate, and Flat as control substrate. Overall, the YAP nuclear/cytoplasm ratio was not changed by either Noco or Bleb treatments (Fig. S4b).

Taken together, these data demonstrate that PC12 cells have an increased YAP nuclear localization (and hence YAP activation) is cultured on NG topographies. However, pharmacological tuning by Noco or Bleb had no major effects on YAP nuclear localization.

#### Comparative test with primary hippocampal neurons

**Figure 8:**
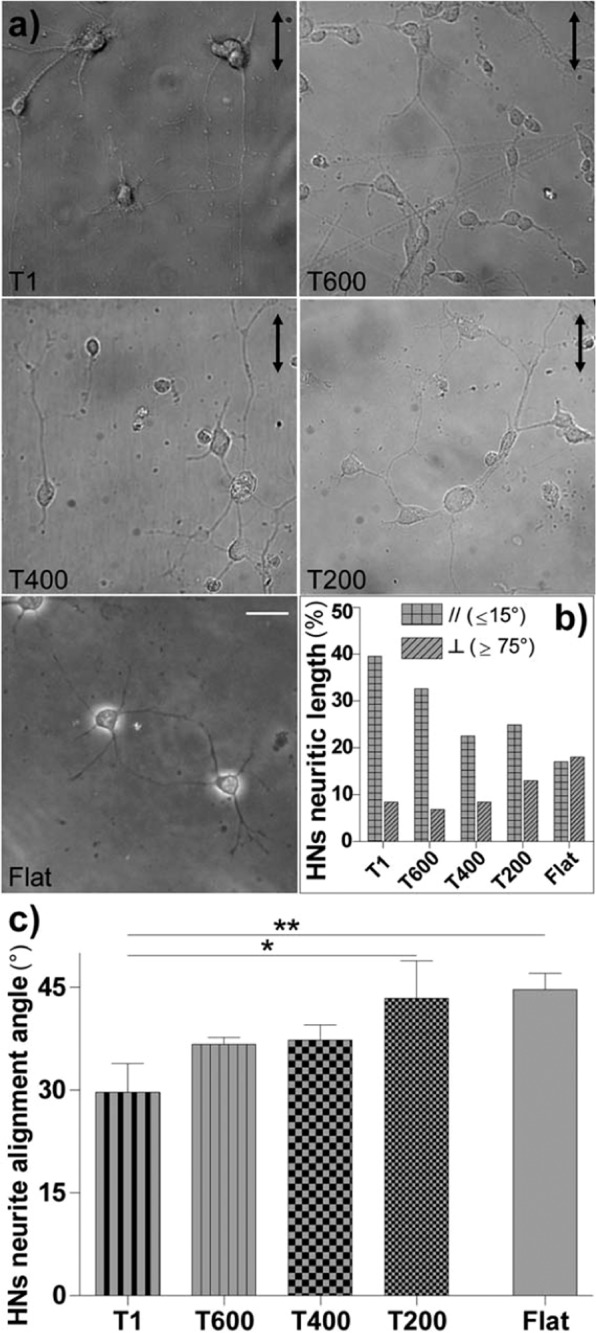
Impact of ultra-small NGs on hippocampal primary neurons (HNs). (**a**) Bright-field images of HNs from wild-type mice on T1, T600, T400, T200 and Flat, at day-*in-vitro* (div) 2; the arrows indicate the NG direction; scale bar = 25 μm. (**b**) HNs network development on ultra-small NGs: we quantified the % of HNs *aligned* network- i.e. the sum of all neurites with angle 0–15° vs. NG direction (*squared columns*)- and the % of *perpendicular* network- i.e. with angle 75–90° vs. NG direction (*striped columns*)- over the total neuronal network analyzed. (**c)** Neurite alignment angle of HNs on ultra-small NGs. *P < 0.05 T1 vs. T200, **P < 0.01 T1 vs. Flat (One-way ANOVA, Tukey’s test). We performed n = 4 independent experiments for T1, and n = 2 independent experiments for T600-T400-T200, with at least 15 neurons analyzed for each substrate; data = mean ± SD.

In order to validate our model, we studied the response of wild-type murine primary hippocampal neurons (HNs) to our ultra-small NGs (Fig. 8a). Bright-field imaging on HNs (div3) showed how the neuronal polarization and neurite network alignment along NG pattern progressively decreased with the reduction of NG dimension (Fig. 8a).

Because we know that neurite growth is usually favored along NGs (i.e. aligned neurites are longer than misaligned ones)^55^, we quantitatively examined the spatial development of neurite arborization and we calculated the total length of the neurites aligned (alignment angle 0–15°) or perpendicular (alignment angle 75°–90°) to NG pattern on each substrate (reported in % over the total network length analyzed) (Fig. 8b). The total neuritic length derived from aligned neurites decreased with NG periodicity, thus maintaining an overall alignment also on T200 if compared to the flat random spatial distribution. On the other hand, the amount of network perpendicular to the pattern had the opposite trend (Fig. 8b). Regarding the mean neurite alignment angle, T1 led, as expected, to the best alignment of the neuronal network (P < 0.01 *vs*. T200 and Flat, Tukey’s test; Fig. 8c), T600 and T400 showed an overall intermediate guidance capability, while T200 showed almost no directional preferential outgrowth of neurites (P < 0.01 T200 *vs*. T1, Tukey’s test), similarly to the Flat control condition (Fig. 8a,c). Overall, ultra-small NGs induced similar neurite guidance effects on both neuro-differentiated PC12 cells and murine primary hippocampal neurons.

## Discussion

The aim of this work was to elucidate the mechanism and the minimum dimensional limit underlying the topographical guidance response of neuronal cells (i.e. NGF-differentiating PC12 cells) to directional NGs, and the role of YAP/TAZ in the nanotopographical guidance process as well. We developed ultra-small NGs with different lateral periodicity, starting from a 1000 nm period (T1), as *best guidance* reference substrate^11,14^, and scaling down to 200 um period (T200). Importantly, our intermediate mold fabrication method allows replicating ultra-small NG structures, preserving the initial mold and obtaining a high replica process yield.

We analyzed the influence of NG topography on YAP, to clarify its role in neuronal contact guidance. The expression of YAP was not influenced by substrate nanotopographies, however its localization (and therefore likely its activation) was. Importantly, YAP localized more in the nucleus of cells cultured on NGs than in that of those on Flat isotropic substrates (Fig. 7c). The nuclear localization of YAP complex has been attributed to different factors, including low cell density^56^, mechanical stretching and substrate stiffness^44^. Here, the cell density (low) was the same on all our NGs, as well as their substrate stiffness (2.5 ± 0.2 GPa^14^), therefore we envision that the YAP nuclear localization is induced in PC12 cells by the NG topography itself, likely *via* cytoskeleton remodeling and mechanical stretching. It is, in fact, well known that NGs activate cell polarization and cytoskeleton via the Rho-mediated pathway^11,14,30^, As reviewed in the introduction, in literature there are only few reports about YAP translocation/activation induced by micro/nano topographies, and with conflicting outcomes. Our results show that all NG topographies (from T1 to T200) activate YAP nuclear translocation with respect to unpatterned control substrates. However, YAP localization is not sensible to the different pattern periodicity, and importantly neither to the contact guidance performance of the cells.

In fact, regarding the neurite topographical contact guidance performance on ultra-small NGs, we found that NGs with periodicity below 400 nm (*i.e*. T200) do not allow proper PC12 neurite contact guidance, and that this is linked to the loss of the FA constraint on NG ridges and to reduced FA maturation. Similarly, neurite alignment to NG patterns decreases with NG downsizing also in murine primary neurons. Regarding the neurite guidance performance on NGs, our group previously demonstrated that neurite contact guidance strictly depends on FAs spatial distribution, density and maturation level^11,29^, In fact, FAs act as topographical sensors and retrieve essential guidance cues, leading to the maturation of aligned FAs and the relative neuritic protrusions, and to the growth-stop/collapse of the misaligned ones. Interestingly, the progressive reduction of NG periodicity correlates with a progressive decrease in the amount of FA alignment and an increase in FA number/cell (Fig. 3). T400, where the neurite guidance is still efficient, behaves as a “border land” substrate, between FA alignment and misalignment regimes. Even if T200 (i.e. where neurite guidance is impaired) maintained an overall good degree of FA alignment (i.e. ~40% of FAs aligned to NG pattern) with respect to a random distribution (i.e. ~17% of FAs aligned to any randomly chosen distribution), on T200 the number of FAs per cell was highly enhanced, towards Flat value. A similar phenomenon (i.e. the increase of FAs/cell number) was also found in PC12 cells neuro-differentiating such as response to the progressive reduction of the substrate directionality signal on “noisy” substrates, and it was proportional to the loss of neurite guidance^11^.

We know that the aligned FAs have the strongest cytoskeleton anchorage and that they are primarily involved in cell tension, as shown in Tonazzini *et al*.^11^. Looking at the FAs size, we found that FA maturation was differently modulated by *neurite-guiding* NGs (T1, T600 and T400) and *less*/*not-guiding* ones (T200 and of course Flat). In fact, the average size of *aligned* FAs was higher if compared to the size of *misaligned* FAs on T1, T600 and also T400 (*limit periodicity for efficient neurite guidance*), while this was not the case on T200 and Flat **(**Fig. 4a). Similarly the amount of large FAs (>2 µm^2^) developing aligned to NGs was highly increased on T1, T600 and T400 substrates, in comparison to the amount of large FAs developing misaligned **(**Fig. 4b). In the case of T200 the FA maturation was independent from their spatial position, and the percentage of aligned and misaligned large FAs was the same. We figure out that on T200 there is no inhibition in the maturation of misaligned FAs, thus leading to the development of aligned and misaligned FAs with similar size, and likely to the increase of FAs number. Here FAs are not confined by ridges and can develop across grooves. We propose that this process leads to the impaired neurite topographical guidance on T200.

Overall, the maturation of aligned FAs on the T200 was reduced. These findings led us to the concept of the minimal critical size of FAs in cells. Some studies in literature sustain the idea that proteins within FAs assemble into higher order structures on the ∼100 nm length scale^57^. Because of the difficulty to fabricate pure physical patterns in the ~100 nm range dimension, most of the studies on FA size reported in literature have been performed on biochemical or nanopillar patterns. Human endothelial cells (HUVEC) showed compromised and no pattern-specific adhesion on fibronectin nanodots smaller than 100 nm^58^. Arnold *et al*. produced adhesive patches of RGD peptides with side lengths ranging from 100 to 3000 nm and showed that on patterns below 500 nm rat embryo fibroblasts (REF) bridged multiple adjacent ligand domains via individual actin fibers in order to adhere^59^. In the case of MDCK epithelial cells cultured on polymeric NOA-61 nanopillars^60^ (diameters ranging from 200 nm to 700 nm), the size of the FAs was confined by the size of the nanopillars. As in our case, the number of FAs increased and the total FA area decreased as the size of nanopillars decreased. However, the FAs formed on the 200-nm nanopillars were larger than those formed on the 400-nm nanopillars. Although the spatial limitations of the nanopillars prevented the maturation of focal complexes to focal adhesions, cells exerted considerable force on the 200 nm-nanopillars, leading to bending of the nanopillars to form larger focal adhesions from adjacent focal complexes. Similarly NIH 3T3 fibroblasts cultured on PMMA nanopillars with a diameter of 70 nm formed very small adhesions, which led to a higher cells motility^61^.

In line with these reports, we found a reduced maturation of FA at molecular level on T200. In order to further investigate the influence of ultra-small NGs on FA activation, we analyzed the activation of FAK and the expression levels of Talin, Vinculin and Zyxin by Western blotting (Fig. 5). FAK is a kinase involved in mechano-signalling pathway of FAs; Talin and Vinculin are proteins of the mechanosensing structural module, connecting FAs to actin cytoskeletal fibers; Zyxin accumulates in the more mature FAs and binds alfa-actinin dimer in the cytoskeleton^9^. We found a reduced level of Zyxin in the cells cultured on T200 NG, while the activation of FAK was not affected by the different NGs, neither Talin nor Vinculin levels. It is noteworthy that Zyxin is one of the FA components that are absent from focal complexes^62^ and are recruited to adhesion sites after application of mechanical stress^63,64^, when FAs enlarge and mature.

We propose that the loss of neurite guidance on T200 stems from a reduced development and maturation of FAs, in particular the aligned ones. Overall, FA development is the crucial mechanism responsible for nanotopography reading process and for the sensing of the reduced dimensionality of NGs, while YAP was secondarily involved.

However, a further contribution to the gradual loss of neurite contact guidance can be the progressive reduction of the directional signal given by the different NGs themselves. Our previous studies^11^ demonstrated that hindering the NG directional signal by nanotopographical noise without altering the periodicity was a strategy to tune the PC12 guidance response^65^. Here, we also quantified the directional signal given to PC12 cells by the different NG periodicity, by FFT analysis. We found that it decreases in intensity with the decrease of the NG periodicity (Fig. 1d); therefore, PC12 perceived a different direction stimulus on the different NGs. This reduced substrate directionality signal may take part in the loss of neurite alignment when the periodicity is downscaled^11^. However, the neurite alignment was still efficient on T400 while it was impaired on T200, although these NGs have similar directionality amplitude signal. We therefore believe that the main factor playing in contact guidance response remains the effect of NG size on FA development.

It is well known in literature that contact guidance requires Rho-associated protein kinase (ROCK)-myosin-II contractility^11^ and that it is modulated by pharmacologically interfering with this signaling pathway^9,11,30^, In order to tune neurite guidance along ultra-small NGs, we investigated the effect of pharmacological treatments targeting cell contractility by Noco and by Bleb^11,66^, Noco, contrary to what was obtained with Bleb, had no effect on NGs with high neurite guidance performance (*i.e*. T1-T400). However, in the case of T200, Noco slightly improved the contact guidance response, thus helping cells to align their neurites to the NG pattern. These results may suggest that the increase in cell contractility induced by Noco would restore the reduced FA maturation on T200, in agreement with previous results induced by Noco in the presence of topographical noise^11^.

Owing to the acto-myosin cytoskeleton role in the transduction of the mechanical signal (which causes YAP/TAZ activation), we also investigated the effects of cytoskeleton drugs to tune YAP system. Bleb has been reported to block YAP/TAZ activation^44^. However, no main differences were measured between the control and treated conditions for YAP sub-cellular localization in PC12 cells, both on selected NGs either on flat substrates (Fig. S4**)**. It is known that the substrate stiffness tightly controls the subcellular localization of YAP^44^, in fact if cells grow on a hard substrates YAP/TAZ complex translocate to the nucleus. Moreover the activation of YAP in human mesenchymal stem cells (hMSCs) cultured on soft PEG hydrogels was dependent on the previous culture time on stiff tissue culture polystyrene (~3 GPa)^67^. Our cyclic olefin copolymer (COC) substrates, both ultra-small NGs and Flat, have a stiffness over 2 GPa^14^, which was above the stiffness value able to activate YAP in other cell types, and it may result in a “basal” YAP nuclear translocation in our experimental model which may partially mask here the action of drugs, in particular on NGs.

Taken together, our results suggest that the loss of neurite guidance with the reduction of NG dimensionality is linked to a release of the FA constraint, probably caused by the loss of a directional maturation of FAs and likely by the FA formation also on the grooved area. On ultra-small NGs neurites maintain the ability to follow the topographical signal in terms of track alignment even when FA alignment starts to be compromised, as showed on the T400. Importantly, all NGs activate YAP translocation into the nucleus, likely via their ability to induce cytoskeleton activation. Overall, the YAP pathway is only partially implicated in neurite contact guidance process and also T200 NGs, with its compromised guidance performance, ensured the activation of YAP nuclear translocation.

## Conclusions

In the present work, we studied the neurite contact guidance performance of PC12 cells on ultra-small NGs, with the aim to investigate the lower limit in dimensionality for proper neuronal contact guidance, and the molecular mechanism mediating this process, at the level of FA and YAP/TAZ pathways. We fabricated ultra-small NGs by a new two-step fabrication process, to replicate NG with periodicities below few hundreds nm with high fidelity and yield. Overall, the T200 NG emerged as the NG where neurite contact guidance was compromised, in terms of neurite alignment, FAs spatial distribution and maturation level. Ultra-small NGs induced similar neurite guidance effects on both neuro-differentiated PC12 cells and murine primary hippocampal neurons. Finally, we found that YAP sub-cellular localization is significantly shifted towards nucleus on all NG patterns with respect to Flat surfaces, suggesting that nanotopograhical guidance response in cells can activate YAP.

## Materials and Methods

### Initial molds

Ultra-small nanogratings (NGs) topographies (i.e. anisotropic patterns of alternating lines of ridges and grooves) were fabricated from initial molds by nanoimprint lithography with the following characteristics: T1 (period 1 µm, depth 350 nm), T600 (period 600 nm, depth 300 nm), T400 (period 400 nm, depth 200 nm), T200 (period 200 nm, depth 100 nm); period = ridge width + groove width. T1 silicon mold was obtained by means of electron-beam lithography (EBL) and reactive ion etching (RIE) starting from commercial p-doped silicon wafer (SYLTRONIX, France), as previously reported^27,30^.

T600 and T400 polymeric molds were fabricated by Laser interference lithography (LIL). A SPR220-1.2: PGMEA solution (2:3) was spun onto a silicon wafer with a spin speed of 4000 rpm for 30 s; SPR220-1.2 photoresist was purchased from Microposit (Shipley European Limited, UK), while PGMEA (propylene glycol monomethyl ether acetate) from MicroChem Corp (Newton, MA, USA). The sample was exposed to a 50 mW helium cadmium (HeCd) laser, emitting a TEM00 single mode at a 325 nm light source, with a beam incidence angle of 166° for T600, and of 24° for T400, and an exposure dose of 77 mJ/cm^2^. The resist developing step was performed by immersing the samples in a MF24A/Milli-Q water (10:1) solution for 20 s^68^. The exposure and process parameters (i.e. beam incidence angle, exposure, developing time) were chosen in order to obtain gratings with a period of 600 and 400 nm, 50% duty cycle.

The silicon T200 mold was purchased from ThunderNIL Srl (Basovizza, Trieste, Italy). Finally flat control molds were obtained by silanization of 2 × 2 cm^2^ flat silicon wafer.

### PerFluoroPolyEther (PFPE) intermediate molds

PFPE resin (FLUOROLINK® MD 700, Solvay Speciality Polymers, Bollate, Italy) was mixed with 3%-wt photoinitiator Darocure 1173 (C_10_H_12_O_2_, 405655 Sigma Aldrich), poured on top of the NGs initial molds, and crosslinked by UV-light (365 nm, 25 mW·cm^−2^). The exposure was performed in two steps, as reported in Masciullo 2018^54^: briefly, the samples were kept for 180 s in nitrogen atmosphere, then for 60 s in air. After curing, the PFPE films were easily peeled off and cleaned with nitrogen flow.

### Cyclic olefin copolymer (COC) replicas

COC foils (thickness 140 µm, Microfluidic ChipShop GmbH, Jena, Germany) were imprinted using an Obducat Nanoimprint 24 system (Obducat, Lund, Sweden) using the PFPE molds. After cleaning with 2-propanol, the COC substrates were placed on top of the molds and softened by raising the temperature up to 150 °C. A pressure of 50 bar was then applied for 300 s before cooling down to 70 °C, i.e. below the glass transition temperature of the copolymer (T_g_ = 134 °C). Finally, the pressure was released and the mold was detached from the imprinted COC with a scalpel.

### Surface characterization

COC replicas were imaged by Scanning Electron Microscopy **(SEM**) after coating them with a 5-nm thick gold layer by thermal evaporation. The metal layer was shorted to the SEM sample holder to avoid electron charging during the imaging characterization. The substrates were then loaded into a LEO 1525 field emission SEM and image acquisition was carried out by secondary-electron detection with the Everheart-Thornley detector in order to enhance the topography of substrates.

NGs replicas were also evaluated by atomic force microscopy (**AFM**) (Veeco Innova Scanning Probe Microscope, Veeco Instruments Inc., Santa Barbara, CA, USA), operating in tapping mode by setting 0.977 Hz scan frequency and scanning areas of 3.5 × 3.5 μm^2^ and 10 × 10 μm^2^ (at least 3 areas per sample and 512 points/line). At least three COC replicas were imaged for each NG topography. A silicon nitride tip with a nominal spring constant in the range of 0.2–0.8 N/m and a resonant frequency of 45–95 kHz was used. All the measurements were performed in air at room temperature and raw scan data were levelled by surface subtraction to remove possible sample tilts. NGs profiles were instead acquired by the use of the Gwiddion software (Gwiddion 2.47 version, “Profile” tool). Data are reported as mean ± SD.

Substrate wettability was evaluated by contact angle measurements acquired with a CAM 200 instrument (KSV Instruments, Helsinki, Finland). A deionized water drop was deposited on top of each substrate through a micro-syringe. To evaluate the wettability in both parallel and perpendicular directions with respect to the NG anisotropic pattern, samples were rotated by 90° and three different measurements were acquired for each direction, for all NGs. However, the direction of the pattern did not influence the contact angle values, therefore data were reported as single values, without specifying the direction of measurement acquisition. All these measurements were performed in air at room temperature. Data are reported as mean ± SD.

### Substrate directionality evaluation

NGs substrate directionality was quantified by analyzing the AFM profiles with the “Signal Processing: FFT” tool of the software Origin (https://www.originlab.com, version 9.0). This plug-in returned a plot of frequency versus signal amplitude by exploiting fast Fourier transform (FFT) algorithms: isotropic images generate a flat histogram, whereas oriented images give a peaked histogram. We selected only the FFT main peak, correspondent to the peculiar NGs periodicity. We analyzed at least 12 profiles (length 10 μm) for each NG image (image dimensions were kept fixed at 10 × 10 μm^2^). We used AFM images of COC replicas for T400 and T600 substrates to extract the NGs profile, while AFM images of the initial silicon mold for T200 substrates, to avoid noisy peaks in the T200 profile caused by electrostatic interactions between the COC foil and the AFM tip. For all conditions (Flat, T600, T400 and T200), FFT signal amplitude values were normalized to the T1 values. Data are reported as mean ± SD.

### Cell cultures and treatments

PC12 cells (CRL-17210, ATCC) were grown in RPMI medium supplemented with 10% HS, 5% FBS, 2 mM glutamine, 10 U/ml penicillin and 10 mg/ml streptomycin (Thermo Fisher, Waltham Massachusetts, USA) and were maintained in standard conditions (37 °C, 95% humidity, 5% CO_2_). Cells (within the 15th passage) were cultured until sub-confluence, then harvested for cell tests, pipetted to obtain a single cell suspension (through a 10 ml syringe with G21 needle), and seeded onto the NGs at a final concentration of 10^4^ cells/cm^2^. Before cell culturing, the imprinted dishes were sterilized by treatment with ethanol and then rinsed twice with H_2_O_mQ_.

For experiments, PC12 were seeded on substrates (50000 cells/cm^2^ or 100000 cells/cm^2^ – for western blot) and neuronal differentiation was induced by treatment with nerve growth factor (NGF), 100 ng/ml. PC12 cells were allowed to adhere for 7–8 h before stimulation with NGF.

For contractility experiments during PC12 differentiation, PC12 were treated with nocodazole (Methyl-[5-(2-thienylcarbonyl)-1H-benzimidazol-2-yl]-carbamate, dissolved in DMSO, 10 nM; **Noco**) or blebbistatin (1-Phenyl-1,2,3,4-tetrahydro-4-hydroxpyrrolo[2,3-b]-7-methylquinolin-4-one, dissolved in DMSO, 25 µM; **Bleb**); DMSO concentration never exceeded 0.5% v/v and the corresponding solvent concentration was added to the untreated condition. Noco, a microtubule destabilizer, was added after 6 h from NGF treatment, while Bleb, an inhibitor of myosin II, was added 30 min before NGF stimulation (as in Tonazzini 2013^11^).

### Neurite guidance imaging and analysis

Living-cell imaging was performed after 24 h from seeding using a Leica CTR 4000 microscope (Leica, Microsystems, Wetzlar, Germany); at least 15 transmission images (40x) were acquired for each sample.

PC12 neurite response to NGs and Flat substrates was quantified by measuring neurite *alignment* along the NG direction, *length* (in µm) and *straightness* (ratio between the distance from the initial and end point of the neurite and its length), at 24 h, in untreated conditions and after drug treatments.

Morphometric data were collected using ImageJ (National Institute of Health, USA). The NG direction was measured as an angle by ImageJ angle tool; FLAT substrates were given a 0 grating angle.

Neurites were semi-automatically segmented (from the point of origin at the perimeter of the cell body to the tip of the neurite growth cone) using NeuronJ, a plugin of ImageJ designed for neurite tracking. The presence of neurites was evaluated and the alignment quantified by measuring the angle of each neurite with the direction of the NG (or with a randomly chosen direction for cells on flat substrate). Only protrusions originating from the cell body and longer than 10 µm (about one average cell body diameter) were counted as neurites. Only neurites which terminated in a free end or with growth cones cleanly abutting neighboring cells were considered. A file containing the tracks was exported and loaded in Matlab (MathWorks) where a custom program calculated the neurite *length* (the distance of the traced neurite path), *straightness* (ratio between the distance from the initial and end point of the neurite and its length) and *alignment* (measured by approximating the neurite as a straight line from the initial to end point and taking the angle of this line versus the NG orientation), for each time point.

We analyzed at least 300 cells (450 neurites, n ≥ 6 independent experiments) for each sample in control conditions, while at least 110 cells (180 neurites, n ≥ 3 independent experiments) for each sample in drug-treated conditions.

### Focal adhesion experiments and live cell Total Internal Reflection Fluorescence (TIRF) microscopy

PC12 cells were transfected with EGFP-Paxillin construct (gift from Juergen Wehland, Helmholtz Centre for Infection Research, Braunschweig, Germany) by electroporation by Neon transfection system (Thermo Fisher, Waltham Massachusetts, USA), as previously reported^11^. Neuronal differentiation was induced by treatment with NGF, and after 24 h (and >16 h from NGF administration), single PC12 cells adhering to the NGs substrates and to flat control substrates were imaged by live cell imaging. Total Internal Reflection Fluorescence (TIRF) imaging was performed using an inverted Leica AF6000 microscope with an oil immersion 100 × 1.46 NA TIRF objective. For each region, two bright-field (focused on the cell and on the nanostructure), an epifluorescence and a TIRF (depth 150 nm) images were acquired.

For FA analysis, TIRF images were loaded into ImageJ and inverted. FAs were manually drawn using the ‘freehand selection’ tool. Then measurements of FA area (in µm^2^) and alignment angle versus NG direction were then obtained using the ‘measurement’ and ‘angle’ tools of ImageJ, respectively. The angle of each FAs was measured with respect to the NG direction choosing the cell soma center as origin; a random reference direction (0°) was chosen for the flat surfaces. FAs were considered *aligned* if the angle is between 0 and 15° and *misaligned* if between 15 and 90°. The number of FAs per cell was also registered. We analyzed at least 30 cells or 400 FAs for each sample, and for each NG type we performed n ≥ 4 independent experiments.

### Western blot

Western blot analysis on PC12 was performed to assess: the activation (phosphorylation) levels of effector proteins in FA pathway, such as focal adhesion kinase (FAK); the expression levels of FA proteins talin, zyxin and vinculin; the expression levels of YAP, looking at YAP1 level.

PC12 were cultured and NGF-differentiated for 24 h on different substrates and lysed on ice by RIPA buffer (Sigma-Aldrich, R0278) containing protease and phosphatase inhibitors cocktail (cOmplete and PhosSTOP, Roche Diagnostics, Basel, Switzerland). Cell lysates were centrifuged (15000 *g* for 15 minutes, 4 °C) and then the supernatants were tested for protein concentration by a protein assay kit (Micro BCA™, Thermo Scientific Pierce). The samples were mixed with Laemmli buffer containing β-mercapto-ethanol (5% final concentration), boiled for 5 minutes, and used for gel electrophoresis (or kept at −80 °C).

Cell lysates (10 µg/line) were processed by immunoblot, as in^55^. Briefly, samples were resolved by gel electrophoresis (SDS-PAGE) using Gel Criterion XT-Precasted polyacrylamide gel 4–12% Bis-Tris (Biorad), transferred to nitrocellulose membranes by Trans-Blot Turbo transfer system (Biorad) and probed overnight at 4 °C with primary antibodies. We used the following antibodies against: FAK (1:1000; Abcam, Cambridge, UK; ab40794) and phospho(Tyr397)-FAK (1:1000; Abcam ab4803); talin-pan 1–2 (1:1000; Sigma clone 8D4); zyxin (1:1000; Abcam Ab71842); vinculin (1:1000; Abcam ab18058); YAP1 (1:1000, Abcam ab39361) and GAPDH (1:3000; Sigma G8795); the antibodies anti-phospho proteins were incubated in BSA 5% buffer, while the others in milk 2%. Membranes, after incubation with the appropriate peroxidase-linked secondary antibodies (goat anti-Rabbit/Mouse IgG-HRP Conjugate, Biorad; 1:2500), were developed by the SuperSignal West Femto (Thermo Scientific Pierce, #34095) or ClarityTM (Biorad, 170-5060) enhanced chemiluminescent (ECL) substrates. The chemiluminescent signal was acquired by ImageQUANT LAS400 scanner (GE Healthcare Life Sciences, Uppsala, Sweden). The density of immunoreactive bands was quantified by ImageJ. The results for each protein were normalized to the relative GAPDH content; the results for pFAK were further normalized to the total FAK protein levels. The results are reported in % with respect to the T1 levels of each gel. For each sample, we performed WB analysis on n ≥ 4 independent experiments.

### YAP immunocytochemistry and fluorescence confocal microscopy

PC12 cells were cultured, fixed for 15 min with 4% formaldehyde with 4% sucrose in PBS at room temperature and processed as previously reported^55^. Here cells were incubated with primary anti-YAP1 antibody (1:500, ab39361) and phalloidin-Alexa fluor 647 (1:40, Thermo Fisher A22287) in GDB buffer (0.2% BSA, 0.8 M NaCl, 0.5% Triton X-100, 30 mM phosphate buffer, pH 7.4) overnight at 4 °C and then with appropriate secondary antibody conjugated to AlexaFluor 488; in the end samples were mounted using Fluorashield mounting medium with 4′,6-diamidin-2-fenilindolo (DAPI) to stain nuclei (Sigma, F6057).

Confocal images were acquired using a laser scanning confocal microscope TCS SP2 (Leica Microsystems) equipped with 63x oil objectives, an argon (488 and 633 nm) laser and a 375 nm laser. All microscope settings were kept constant during sample imaging.

Each confocal image (1024 × 1024 pixel resolution) was obtained from a *z* -series (stack depth was within 10 μm; steps = 1 μm; each image was averaged three times between frames) and was chosen to cover the entire region of interest from top to bottom. The resulting *z* -stack was processed by ImageJ software into a single image using “z-project” and “max intensity” options. The confocal settings were kept the same for all scans when fluorescence intensity was compared.

The confocal images of actin fibers and DAPI staining were used to evaluate *cellular* and *nuclear* YAP localization levels by ImageJ (“polygon selection” tool for cell contours, while “make a binary” and “wand tool” for nucleus contours). The YAP signal was quantified as follows: the cell or nucleus ROI were applied to the correspondent YAP positive images and their intensity were measured by the ImageJ “Measure” tool (option “*mean grey value”*). The values were then reported as the nuclear/cellular ratio; at least 25 cells/sample were analyzed, and for each NG type we performed n ≥ 3 independent experiments.

### SEM imaging

PC12 cells cultured on NGs substrates and fixed were processed and metallized for scanning electron microscopy (SEM) as previously reported^23^. The substrates were then loaded into a LEO 1525 field emission SEM and image acquisition was carried out by secondary-electron detection with an Everheart–Thornley detector in order to enhance the topography of cell–substrate interfaces.

### Primary hippocampal neurons

Hippocampal neuronal cultures (HNs) were prepared from brains of single E17–18 mouse embryos (C57 background), as previously described^55^. Mice were maintained under standard housing conditions and used according to the protocols and ethical guidelines approved by the Italian Ministry of Health (Permit Number: CBS-not. 0517; approved the 4/1/2018, at CNR- Pisa) and in accordance with the European Commission Council Directive 2010/63/EU. Pregnant dams were sacrificed by cervical dislocation, while embryos were decapitated with surgical scissors. In brief, the hippocampi were removed from the brains, incubated in trypsin/EDTA solution, re-suspended in neurobasal medium supplemented with 2% B27, 1% penicillin/streptomycin, and 1% glutamax (Invitrogen), and dissociated using a gently flamed Pasteur pipette. HNs were plated on poly-L-lysine- (37.5 µg/ml; P4832 Sigma) and laminin-coated (2.5 µg/ml, L2020 Sigma; in 0.1 M borate buffer pH 8.5) NGs and flat substrates at a density of 50000 cells/cm^2^. At day-*in-vitro* (div) 3, bright-field cell imaging was performed using an inverted wide-field microscope Eclipse *ti* (Nikon, Japan), using an oil 40 × 1.3 NA objective (Plan-Fluor, Nikon, Japan); at least 6 images were acquired for each sample. Only neurons fully visible and with soma cleanly abutting neighbouring cells were traced and analysed. Neurites were semi-automatically segmented by NeuronJ, according to their branching, as in^69,70^. Briefly, neurite segments were divided in categories: *1° order* for neurites directly emerging from the cell soma (from soma to the tip); *2° order* for neurites emerging from 1° order neurites. We focused our analysis on 1° order neurites, being them the vast majority of total neuritic segments at early stages^55,69^. Neurite analysis was then performed such as for PC12 cells, as reported above. HNs neurites (minimum threshold length ≥ 10 µm) were considered *aligned* if their alignment angle is between 0 and 15°, and *perpendicular* if between 75 and 90°, as in^55^. The *total aligned/perpendicular neuritic length* (reported in % over the total neurite network length) were then calculated. At least 15 neurons were analyzed for each sample; results are reported as mean ± SD, from n = 4 independent experiments for T1 and n = 2 for T600-T400-T200.

### Statistical analysis

Data are reported as average value ± the standard error of the mean (mean ± SEM), if not differently stated. All the cell experiments were repeated at least three times independently for each reported dataset (n ≥ 3). Data were statistically analyzed by GraphPad PRISM 5.00 program (GraphPad Software, San Diego, CA, USA). For parametric data (after Shapiro-Wilk normality test), Student’s *t*-test (unpaired, two-tailed) or One-Way ANOVA (Tukey’s or Dunnett’s multiple comparison test) analysis were used; the mean values obtained in each repeated experiment were assumed to be normally distributed about the true mean. Statistical significance refers to results where P < 0.05 was obtained.

## Supplementary information

Supplementary information 
