# Supplementary material for: Neuronal contact guidance and YAP signaling on ultra-small nanogratings
Source: Sci Rep. 2020 Feb 28;10:3742. doi: 10.1038/s41598-020-60745-5 (PMC7048778; doi:10.1038/s41598-020-60745-5)
Supplement: Supplementary file 1 — Supplementary information [file 41598_2020_60745_MOESM1_ESM.docx]

**Neuronal contact guidance and YAP signaling on ultra-small nanogratings**

Ilaria Tonazzini^1^, Cecilia Masciullo^1^, Eleonora Savi^1^, Agnese Sonato^2^, Filippo Romanato^2^ and Marco Cecchini^1,^*

^1^ NEST, Istituto Nanoscienze-CNR and Scuola Normale Superiore, Piazza San Silvestro 12, Pisa 56127, Italy

^2^ CNR-IOM, Area Science Park, S.S. 14, km 163.5, Basovizza (TS), Italy.

**Supplementary**


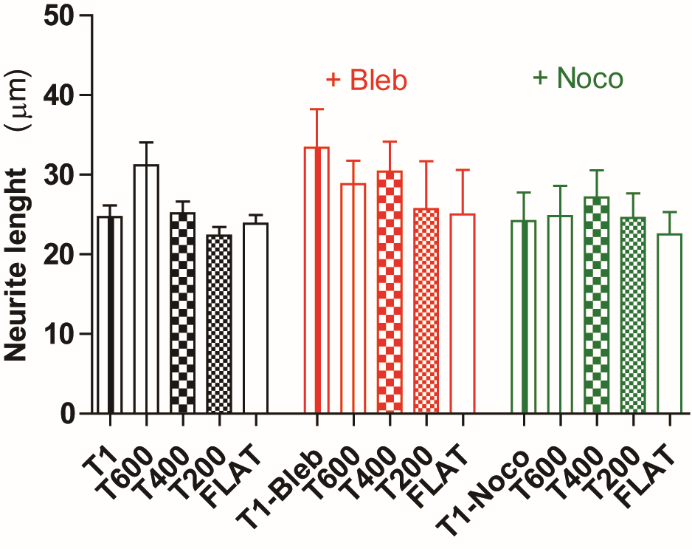


**Figure S1:** Neurite length on ultra-small NGs in control condition (*black*) and in the presence of Bleb (25 µM; *red)* or Noco (10nM; *green*). No main differences have been registered in neurite length. Data = mean ± SEM.


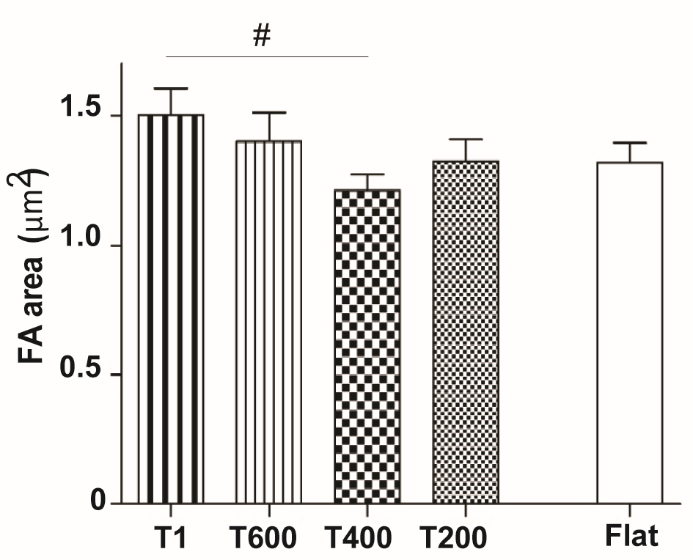


**Figure S2**: Impact of reduced substrate dimensionality on the average FA size (µm^2^). # P < 0.05 T1 *vs*. T400, Student’s t-test. Data = mean ± SEM.


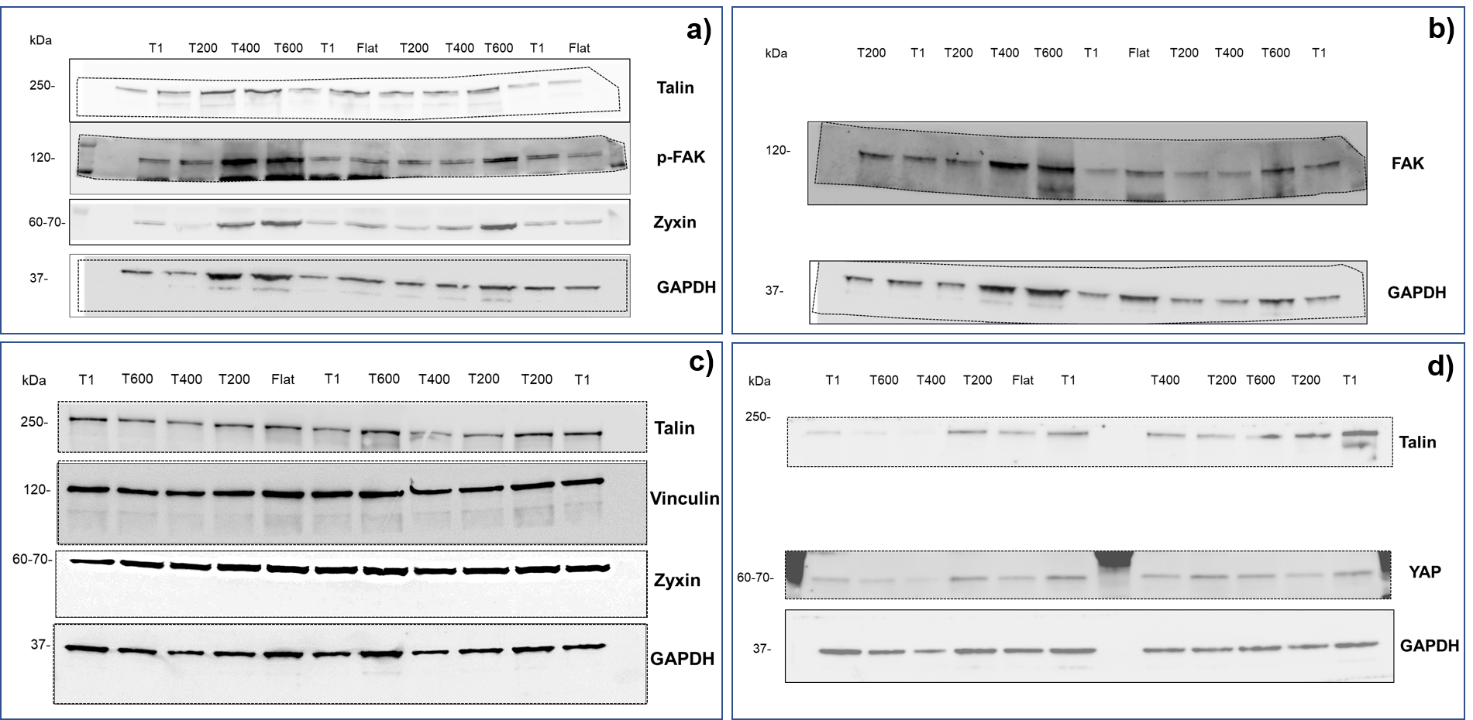


**Figure S3**: a-d) Representative images of some full-length Western-blot membranes (*each panel represents a single gel, cut in different parts*). For each gel, 2 full sets of samples (T1, T600, T400, T200, Flat) were run and processed together. Each membrane was cut in different parts (*dotted lines*), following the molecular mass ladders, and then the different parts were incubated with different antibodies (accordingly to the correspondent protein weight). For pFAK (**a**) and FAK (**b**) proteins, the same lysate samples were run on two different gels in parallel and processed with the two different antibodies (each lane was normalized to the correspondent GADPH levels and then for each sample the results of pFAK were normalized to the total FAK protein levels). The final results were then reported in % with respect to the T1 levels of each gel.

**
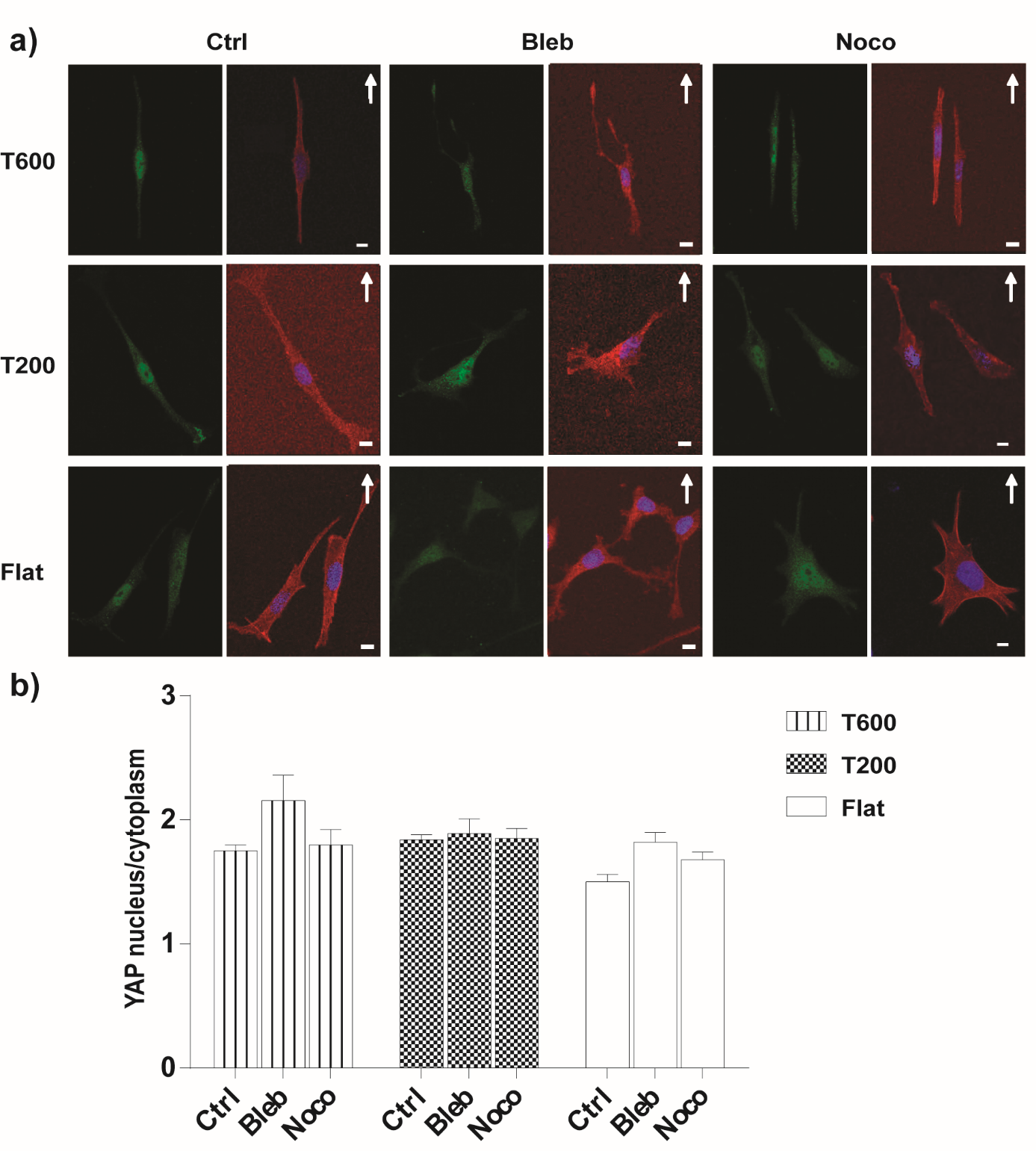
**

**Figure S4:** Effect of contractility targeting drugs on the activation of the YAP pathway. **a)** Confocal representative images of YAP (*green, first column*) and actin fibers (*red*) with nuclear (*blue*) staining in PC12 cells on T600, T200 and Flat substrates in control conditions and after treatment with Blebbistatin (25 µM) and Nocodazole (10 nM); scale bars =10 $\mu$m. **b)** YAP intracellular localization after drug treatments: YAP activation is reported as YAP nuclear/ cytoplasmic ratio on T600, T200 and Flat; at least 25 cells were analyzed for each sample (n ≥ 3), for both control and treated conditions.
